# A natural mineral supplement provides relief from knee osteoarthritis symptoms: a randomized controlled pilot trial

**DOI:** 10.1186/1475-2891-7-9

**Published:** 2008-02-17

**Authors:** Joy L Frestedt, Melanie Walsh, Michael A Kuskowski, John L Zenk

**Affiliations:** 1 Clinical Affairs Department, Minnesota Applied Research Center, Edina, Minneapolis, USA; 2 Marigot Ltd. Co. Cork, Ireland; 3 Department of Psychiatry, Geriatric Research Education and Clinical Center, Minneapolis, USA

## Abstract

**Background:**

This small, pilot study evaluated the impact of treatment with a natural multi-mineral supplement from seaweed (Aquamin) on walking distance, pain and joint mobility in subjects with moderate to severe osteoarthritis of the knee.

**Methods:**

Subjects (n = 70) with moderate to severe osteoarthritis of the knee were randomized to four double-blinded treatments for 12 weeks: (a) Glucosamine sulfate (1500 mg/d); (b) Aquamin (2400 mg/d); (c) Combined treatment composed of Glucosamine sulfate (1500 mg/d) plus Aquamin (2400 mg/d) and (d) Placebo. Primary outcome measures were WOMAC scores and 6 Minute Walking Distances (6 MWD). Laboratory based blood tests were used as safety measures.

**Results:**

Fifty subjects completed the study and analysis of the data showed significant differences between the groups for changes in WOMAC pain scores over time (p = 0.009 ANCOVA); however, these data must be reviewed with caution since significant differences were found between the groups at baseline for WOMAC pain and stiffness scores (p = 0.0039 and p = 0.013, respectively, ANOVA). Only the Aquamin and Glucosamine groups demonstrated significant improvements in symptoms over the course of the study. The combination group (like the placebo group) did not show any significant improvements in OA symptoms in this trial. Within group analysis demonstrated significant improvements over time on treatment for the WOMAC pain, activity, composite and stiffness (Aquamin only) scores as well as the 6 minute walking distances for subjects in the Aquamin and Glucosamine treatment groups. The Aquamin and Glucosamine groups walked 101 feet (+7%) and 56 feet (+3.5%) extra respectively. All treatments were well tolerated and the adverse events profiles were not significantly different between the groups.

**Conclusion:**

This small preliminary study suggested that a multi mineral supplement (Aquamin) may reduce the pain and stiffness of osteoarthritis of the knee over 12 weeks of treatment and warrants further study.

**Trial registration:**

ClinicalTrials.gov number: NCT00452101.

## Introduction

Osteoarthritis (OA), also called degenerative joint disease, is a slow destructive process of the joint affecting millions of people worldwide. Although the exact biochemical cause of OA remains unknown, the process usually begins when the joint structures are abnormal or the stress placed on the joint surfaces is unusually high. The secondary inflammation due to progressive articular destruction appears to be localized to the particular joint being affected. Current anti-inflammatory treatments for OA while providing some relief from symptoms are suboptimal and the side effects associated with these treatments; in particular the COX-2 specific NSAID's are becoming increasingly recognized [1,2]. As a result of this, use of alternative treatments and complementary medicines are gaining popularity in the United States among OA sufferers.

Glucosamine, a structural component of cartilage, is recognized as a nutritional supplement by the US FDA but as a pharmaceutical product in most European and Scandanavian countries as well as some Asian and Latin American Countries. Glucosamine has been the subject of many trials [3,4] and is used worldwide as an "alternative" treatment for OA although the extent to which it may provide relief to the symptoms of OA is still unclear. The recent NIH funded Glucosamine/Chondroitin Arthritis Intervention Trial (GAIT) tested the efficacy of glucosamine in providing relief for subjects with symptomatic knee osteoarthritis [5]. In this multi-centre study, glucosamine hydrochloride was tested either alone or in combination with chondroitin sulfate, a gycosaminoglycan that is also a structural component of cartilage and a popular alternative therapy for OA. In this study, the overall group of subjects failed to demonstrate an improvement in symptoms for both the individual and combined treatments possibly as a result of the large (60%) placebo effect observed. Some benefit was observed in a subset of subjects with moderate to severe knee osteoarthritis suggesting that the benefits of these nutraceuticals may be limited to this group.

In addition to glucosamine and chondroitin, other nutraceutical products have been reported to provide relief from OA [6-9]. Cat's claw extract has recently been combined with a mineral based treatment (Sierrasil^®^) to provide symptomatic relief for a group of mild to moderate OA sufferers. While initially demonstrating some benefit with the cats claw/mineral supplement, Miller and co-workers observed that this benefit was at best temporary for a 1–2 week period [10]. Even though the positive effects were short lived in this subset of OA subjects, growing evidence suggests that minerals may play a role in joint health.

Naturally occurring minerals such as magnesium, copper, manganese, selenium and zinc have shown anti-inflammatory effects in both animal and human studies. In a rat model of osteoarthritis, a deficiency of dietary magnesium was demonstrated to enhance the amount of cartilage damage [11]. Furthermore, increased magnesium in the diet may influence inflammation through reducing the serum level of the pro-inflammatory protein C-reactive protein [12]. The trace element copper is an essential cofactor in enzymes such as the collagen cross-linker lysyl oxidase and the anti-oxidant enzyme super oxide dismutase (SOD) that also requires zinc and manganese as cofactors. Recent evidence has suggested a role for oxidative stress in the pathogenesis of OA whereby an excess of reactive oxygen species arising from an imbalance in the antioxidant status of the joint (such as reduced levels of SOD) may result in cartilage degradation and joint remodeling [13]. Selenium is also an essential co-factor for glutathione peroxidase may have a role in reducing the incidence of osteoarthritic lesion [14,15] Positive roles have also been suggested for trace minerals such as boron and manganese in reducing the symptoms and slowing the pathogenesis of OA [16].

The present study was designed to evaluate the potential for a seaweed-derived multi-mineral supplement to alleviate OA symptoms. The mineral supplement (Aquamin) is derived from the red algae Lithothamnion corallioides which is rich in calcium and magnesium and has a variety of trace minerals (Table 1). The goal of this pilot trial was to gain preliminary data regarding the impact of Aquamin, Glucosamine Sulfate, the combination of Aquamin and Glucosamine Sulfate, or Placebo on symptoms and functional abilities of subjects with OA during 12 weeks of treatment.

**Table 1:** Typical Mineral Composition of Aquamin

**Mineral**	**Dry Salt Weight**
Calcium Carbonate	85% (34% calcium)
Magnesium Carbonate	8.5% (2.4% magnesium)
Salt (as chloride)	1.5%
Moisture	3.0%
Trace Minerals**	2.0%
Sulphur	0.7%
Potassium	0.6%
Phosphorus	0.05%
Sodium	0.25%
Manganese	100 ppm.
Zinc	20 ppm
Iron	800 ppm
Iodine	30 ppm
Boron	17 ppm.
Copper	8 ppm.
Cobalt	0.1 ppm.
Selenium	1.0 ppm.

**Aquamin contains a wide spectrum of trace minerals assimilated from sea water of which the minerals outlined in the remainder of the table are a selection.

Aquamin is a natural ingredient and trace mineral levels may vary over time

## Materials and methods

### Study design

This study was a randomized, double blind, placebo controlled clinical trial with four parallel treatment groups: Aquamin, Glucosamine Sulfate, Aquamin plus Glucosamine Sulfate and Placebo. This trial was performed in compliance with all applicable regulations and guidelines (e.g. International Conference on Harmonization Good Clinical Practices, ICH-GCP, the Declaration of Helsinki, 21CFR50-Protection of Human Subjects, and 21CRF56-Institutional Review Boards) and was approved and continuously reviewed by the Quorum Institutional Review Board (Seattle, WA).

### Sample size

Since pretrial information about Aquamin was entirely anecdotal, traditional effect sizes and sample size estimates were not possible. We used our experience from a previous study with similar endpoints to estimate the number of subjects that might be needed for this pilot trial. In our earlier trial, we found that 6 weeks of treatment with glucosamine sulfate and/or hyper-immune milk had a significant impact on WOMAC pain, stiffness and activity scores among 35 OA subjects with 10–13 subjects in each of the 3 treatment arms [6]. Based on this information, we enrolled 15 subjects in each of the four treatment arms (Aquamin, Glucosamine Sulfate, Aquamin plus Glucosamine Sulfate and Placebo) for a total of 70 subjects enrolled in this small pilot trial.

### Subjects

This was a single centre study conducted at the Minnesota Applied Research Centre and subjects were recruited by advertising in the Minneapolis, Minnesota area. Subjects of either gender were included if they voluntarily gave informed consent, were ambulatory, 25–75 years old, with normal digestion and absorption, diagnosed with moderate to severe OA of the knee according to their previous medical history and the modified clinical criteria of the American College of Rheumatology [17] and had a Western Ontario and McMaster Universities (WOMAC) Osteoarthritis Index [18] score ≤ 75 in the target knee. The target knee was chosen by physical examination to identify the most severely effected knee for each subject and the cut off point for the WOMAC score was enforced as a means of standardizing the extent of pain and immobility in the small number of subjects recruited for this trial. In order to establish a standardized calcium intake across all treatments, subjects were asked to consume a diet with ~600 mg calcium (e.g. two dairy servings) which was estimated to be 40–60% of their RDI (depending on age) per day.

Exclusion criteria were rheumatoid arthritis, gout, pseudogout, Paget's disease, seizure disorder, insulin dependent diabetes mellitus, uncontrolled hypertension, unstable cardiovascular disease, active hepatic or renal disease, active cancer and/or HIV infection or if they required prescription drugs to control pain; had other clinical trial or experimental treatments in the past 3 months; were pregnant, lactating, or at risk of becoming pregnant; or if they received NSAIDS within 48 hours; intramuscular/systemic corticosteroid injection within 4 weeks; intra-articular corticosteroid injection within 2 months; or inter-articular hyaluronic acid injection within 4 months prior to enrollment.

Each subject received one bottle of 350 two-piece hard shell test article capsules each month. Each bottle (and the capsules inside) appeared identical. Subjects were randomized in blocks of 4 using sequential treatment assignments prepared by the independent consulting statistician. The clinical investigator, statistician, clinic staff and subjects remained blinded throughout the trial to avoid bias. The sequence of the study began with a two week period when subjects were asked to discontinue any prescription or over-the-counter or alternative therapy treatments for osteoarthritis.

At the baseline visit, vital signs were assessed and laboratory tests were performed. Subjects were assessed for WOMAC parameters and a 6 minute walking test was performed. After each month of treatment (at 4, 8 and 12 weeks) the subject's diaries, WOMAC questionnaires, and unused pills were collected, medications/supplements were reviewed, adverse events investigated, vital signs measured, blood was drawn and 6 MWD and WOMAC were measured. Active treatment was completed at week 12 when laboratory tests were repeated. Each subject returned to the clinic at 16, 20 and 24 weeks after their treatment began for monitoring of blood chemistry only. Although there was no reason to expect any beneficial carry over effect on the OA symptoms after removal of the treatments, subjects returned to the clinic every 4 weeks for 12 weeks after termination for blood chemistry readings in order to ensure that there were no adverse consequences on their blood metabolites including their blood calcium levels.

### Treatments

The duration of treatment was 12 weeks, administered as three capsules taken with a glass of water, three times per day. The capsules contained Aquamin (267 mg Aquamin F + 167 mg maltodextrin – FCC, USP & NF specifications for 10 Dextrose Equivalent Maltodextrin which was assumed inert in the capsules) designated A in the results section; Glucosamine sulfate (167 mg D-glucosamine sulfate Potassium salt, Pharmachem Labs NJ, USA + 267 mg maltodextrin) designated GS, Aquamin and Glucosamine (267 mg Aquamin F + 167 mg glucosamine sulfate) designated G+A, or Placebo (434 mg maltodextran) designated PBO in the results section. The rescue medication was acetaminophen, 325 mg, 1–2 tablets every 4–6 hours as needed for intractable pain.

### Study measurements and statistical analysis

Joint symptoms were assessed using the Western Ontario and McMaster Universities (WOMAC) Osteoarthritis Index, a validated questionnaire including scores for pain, stiffness and activities as well as a composite (total) score. The WOMAC scores were transformed according to the standard orthopedic formula:

Transformed Score = 100 - (Actual Raw Score × 100/Possible Raw Score) [18]

The values represent "percentage of normal," such that increasing scores reflect improvement and decreasing scores reflect worsening of symptoms. The six minute walking distance (6 MWD) was conducted by marking off a 100-foot distance in an interior hallway and asking subjects to walk as far as they can as quickly as they can over 6 minutes. The total distance was measured and recorded. Adverse effects were assessed by a questionnaire and vital signs/laboratory measurements respectively.

This study was conducted, monitored and audited in compliance with ICH-GCP guidelines and according to the Minnesota Applied Research Center Standard Operating Procedures (SOP's) and the SOP's of certified vendors (e.g. for WOMAC scoring). Subject compliance was assessed at each visit by pill count, interview, and review of the medication diary. Subject data was kept confidential and study records were stored in a locked and secure storage area. An independent statistician used ANOVA for between group comparisons at baseline. ANCOVA (with baseline score as co-variate) were used to assess between-group differences in change over time. Matched pair T-tests were used for within group comparisons of change over time. Data was analyzed under Intent To Treat Last Observation Carried Forward (ITT-LOCF) case condition and statistical significance was set at p < 0.05.

## Results

### Baseline characteristics

Table 2 shows that all four groups were comparable (on average) for number of subjects (N = 15–20), gender (6–11 male; 7–11 female), BMI (30.5 – 32.5), age (58.5 to 60.3 years), WOMAC activity (49.4–63.0) and composite (48.8–63.4) scores and 6 MWD (1323–1427 feet) indicating that the randomization was effective for those parameters. Significant baseline differences were observed between the four groups for WOMAC pain (50.0–67.2) and stiffness scores (40.4–57.8) (p = 0.039 and 0.013 respectively). (Table 3) The finding of baseline differences for the pain and stiffness sub-scores limits the analysis of these data.

**Table 2:** Baseline characteristics (ITT analysis).

**Characteristic**	**N started (completed)**	**Gender M/F**	**Mean age (S.D.)**	**BMI (calc)**
**PBO**	16(9)	6/10	58.9(7.4)	32.4
**A**	20(15)	11/9	58.5(12.1)	32.5
**GS**	19(14)	8/11	59.2(8.3)	32.1
**G+A**	15(12)	8/7	60.3 (9.8)	30.5
p (ANOVA*)	NS	NS	NS	NS

* = ANCOVA comparison between groups

**Table 3:** Changes in WOMAC Scores at baseline and at the end of the trial Between and Within Groups (ITT-LOCF)

**Variable**	**Pain** Baseline	**Pain** End	**Pain baseline – pain end**	**Stiffness** Baseline	**Stiffness** End	**Stiffness baseline – stiffness end**	**Activity** Baseline	**Activity** End	**Activity baseline – activity end**	**Composite** Baseline	**Composite** End	**Composite baseline – composite end**
**PBO**	**50.0**	**52.9**	**-2.9**	**40.4**	**46.3**	**-5.9**	**49.4**	**56.4**	**-7.0**	**48.8**	**54.8**	**-6.1**
**SD**	22.9	21.4	19.9	17.4	25.3	18.3	23.1	24	18.4	21.8	22.7	17.6
Sig.*			NS			NS			NS			NS
**A**	**56.8**	**74.3**	**-17.5**	**44.4**	**65**	**-20.6**	**58.8**	**72.4**	**-13.5**	**57.3**	**72.2**	**-14.9**
**SD**	15.8	17.6	22.7	17	17.5	26.1	16.2	16.8	21.3	15.3	16.6	21.4
Sig.*			.0.003			0.002			0.01			0.006
**GS**	**60.6**	**72.9**	**-12.6**	**51.3**	**61.8**	**-10.5**	**60.1**	**70.7**	**-10.6**	**59.4**	**70.2**	**-10.9**
**SD**	14.8	17.6	16.3	13.8	19.8	24.0	13.9	18.4	15.4	13.2	17.6	15.6
Sig.*			0.003			NS			0.008			0.007
**G+A**	**67.2**	**69.1**	**-1.9**	**57.8**	**64.1**	**-6.3**	**63**	**68.6**	**-5.6**	**63.4**	**68.3**	**-4.9**
**SD**	13.3	16.9	14.0	15.7	17.6	17.7	8.7	13.1	11.0	8.1	12.9	101
Sig.*			NS			NS			NS			NS
**p(ANCOVA)**	0.039#		0.009	0.013#		NS	NS#		NS	NS#		NS

*Sig. = significance by within group paired t-test; 2-tailed; ANCOVA comparison between groups with baseline measure as covariate.

# = ANCOVA comparison between groups showing any differences at baseline for these parameters.

### Subject attrition after release of the test article

**Figure 1:**
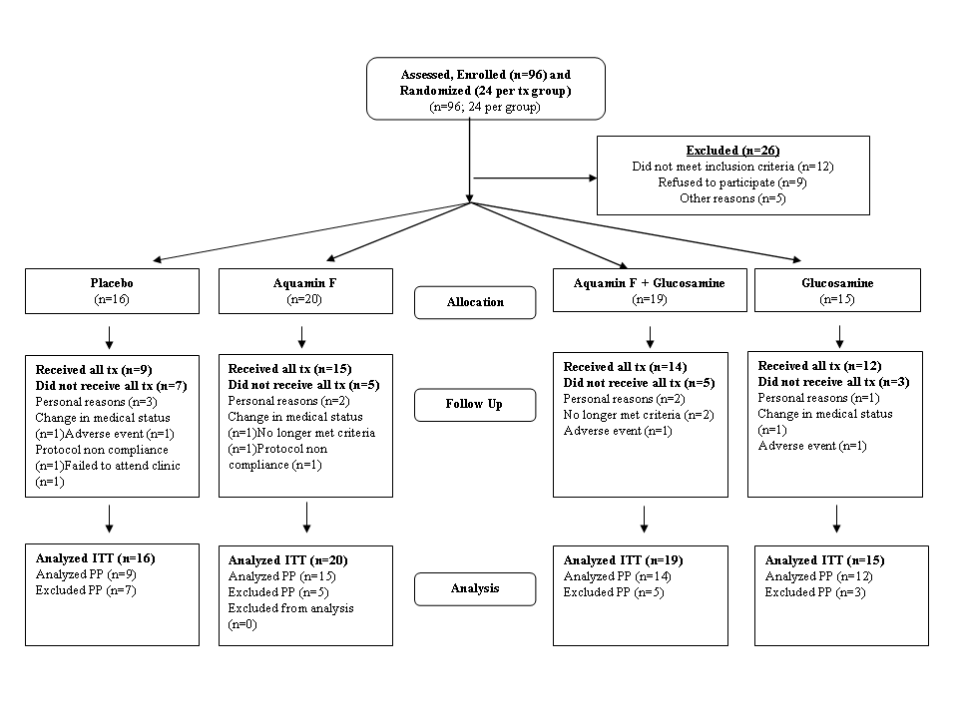
Trial flow chart.

Twenty (20) of the 70 subjects given test article withdrew from the trial prior to completion (29% attrition) and a total of 50 subjects completed the trial (Figure 1). The reasons for subject attrition were spread evenly across the test article administration groups and no pattern was obvious that might suggest withdrawal due to a problem specific to any of the test articles in this trial.

### WOMAC

Using an ITT-LOCF analysis, only the improvements in WOMAC pain score differed significantly between the groups over the course of the study (p = 0.009 ANCOVA).

**Figure 2:**
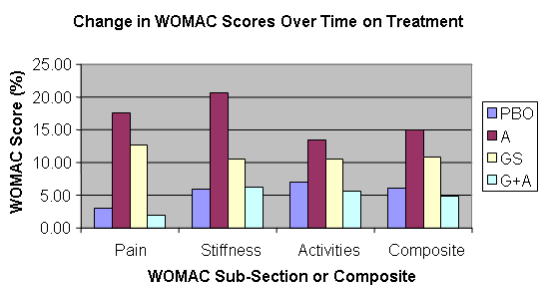
Percent change in WOMAC scores from baseline over 12 weeks of treatment.

All four groups displayed numerical improvements from baseline to end of treatment for WOMAC scores (Table 3 and Figure 2); however, no significant improvements were demonstrated within groups over time for the placebo or for the combined treatment groups. Within group analysis over time showed that the pain score was significantly improved by 17.5 (P = 0.003 ANOVA) for A and 12.6 (P = 0.003 ANOVA) for GS compared to non significant changes of 2.9 for PBO and 1.9 for G+A (higher score indicates less pain).

Within group analysis over time showed that the activity score was significantly improved by 13.6 (P = 0.010 ANOVA) for A and 10.6 (P = 0.008 ANOVA) for GS compared to non significant changes of 7.0 for PBO and 5.6 for G+A.

Within group analysis over time showed that the composite (total) WOMAC score was significantly improved by 14.9 (P = 0.006 ANOVA) for A and 10.8 (P = 0.007 ANOVA) for GS compared to non significant changes of 6.1 for PBO and 4.9 for G+A.

Of interest, the Aquamin group also displayed a significant improvement over time for stiffness score (20.6, p = 0.002) compared to non significant changes of 10.5 for GS, 5.9 for PBO and 6.3 for G+A.

### Subject consumption of rescue medication

No significant differences were found between the groups in the amount of rescue medication consumed over the course of the experiment (Table 4).

**Table 4:** Consumption of rescue medication.

**Group**	**Month 1**	**Month 2**	**Month 3**	**Total**
**Placebo**	39(38)*	27(27)	30(33)	89(88)
**A**	45(62)	19(22)	23(31)	87(96)
**GS**	25(20)	29(38)	31(50)	84(103)
**G+A**	26(55)	26(34)	27(46)	63(112)

*Mean (SD). Group comparisons of rescue medication were non-significant (Kruskal-Wallis one-way non-parametric analysis of variance) at all time periods and for total medications

### Six minute walking distance (6 MWD)

The distance covered during a 6 minute timed walk was significantly improved over time on treatment within the Aquamin group (+101 feet, p = 0.001, Table 5). The glucosamine group also demonstrated a significant improvement in 6 MWD over time on treatment (+56 feet, p = 0.030). No significant improvements were demonstrated within groups over time for the placebo group and surprisingly for the combined treatment group.

**Table 5:** Changes in 6 MWD Between and Within Groups Over 12 weeks of treatment.

**Variable**	**Baseline 6 MWD**	**End 6 MWD**	**Baseline-End 6 MWD**
**PBO**	**1323.4**	**1331.9**	**-8.4**
**SD**	226.1	250.2	109.3
Sig.*			NS
**A**	**1427.5**	**1528.8**	**-101.3**
**SD**	225.6	252.3	121.5
Sig.*			0.001
**GS**	**1410.1**	**1456.8**	**-55.7**
**SD**	246.1	256.1	103
Sig.*			0.03
**G+A**	**1363.1**	**1378.1**	**-15**
**SD**	253.7	253	126.6
Sig.*			NS
**p (ANCOVA)**			NS

*Sig. = significance by within group paired t-test; 2-tailed; ANCOVA comparison between groups with baseline measure as covariate

### Adverse effects

All treatments were well tolerated. Table 6 shows that a total of 51 of the 70 subjects given test product (TA) reported eighty-eight (88) adverse effects (AE) but only 7 of the 88 AE (8%) were considered at least possibly related to the TA treatment. These AE were distributed somewhat evenly across the groups: 1 on PBO, 3 on A, 2 on GS and 1 on G+A and none were considered definitely related to the TA (Table 6). Most of the adverse effects (31/88; 35%) were related to musculoskeletal complaints and these were mainly reports of increased knee pain (n = 19/88; 22%). All AE completely resolved or returned to baseline.

**Table 6:** Adverse effects

**Item**	**Total**	**GS**	**A**	**PBO**	**G+A**
**Number of Subjects with AE**	51	12	12	14	13
**HEENT**	25	6	8	6	5
**Respiratory/Pneumonia**	1	0	0	0	1
**Cardiovasular/Hypertension**	1	0	0	0	1
**Gastrointestinal**	17	5	3	6	3
**GU/Reproduction**	3	1	1	0	1
**Neurological**	5	1	3	1	0
**Dermatological (hives, cat bite)**	2	1	0	0	1
**Musculoskeletal**	31	4	9	10	8
**Increased Knee Pain**	19	4	5	4	6
**Other**	6	1	1	0	4
**TOTAL**	88	18	25	23	22

## Discussion

This trial was designed as a preliminary pilot trial to investigate the potential of a marine derived multi-mineral supplement to reduce symptoms of moderate to severe knee osteoarthritis. The dose of the mineral supplement Aquamin was determined based on previous anecdotal experience and a rigorous Intent to Treat – Last Observation Carried Forward statistical analysis was used to compare the four treatment groups: Aquamin, Glucosamine Sulfate, Combination of Aquamin and Glucosamine Sulfate, or placebo.

These results were confounded because the WOMAC pain scores were significantly different between the groups at baseline and, therefore, these improvements need to be viewed with caution because further study is warranted. In general, significant differences were found between groups for pain scores after 12 weeks of treatment. Within groups over time, the Aquamin treatment group showed significantly improved WOMAC pain, stiffness, activity and composite scores over the course of the 12-week treatment. The glucosamine sulfate treatment group also showed significant improvements over time on treatment for the pain, activities and composite scores (but not for the stiffness scores); however, no significant improvements were found over time on treatment for subjects in the Placebo group or, surprisingly for subjects in the Combination treatment group.

No baseline differences were observed among the four groups for the 6 minute walking distances. Over time on treatment, the Aquamin and glucosamine groups demonstrated significant improvements in 6 minute walking distances (101 feet, p = 0.001 and 56 feet, p = 0.030, respectively). This was an improvement of 7% and 3.5% respectively over their baseline walking distances. No significant differences were found for the walking distances measured for the placebo group and surprisingly for the combined treatment group. Although, these distances appear to be small, our subjects with severe OA indicated that the ability to walk even a little bit further was important to them.

The main limitations of this study were its short duration (12 weeks), lack of assessment for remnant effects after treatment stopped and limited sample size (15 subjects per treatment arm). Glucosamine sulfate has been shown to provide a benefit over a longer course of treatment [4] and its efficacy may have been under demonstrated within this 12 week study period. Additional study of longer treatments in a greater numbers of subjects would be helpful to verify the treatment effect for Aquamin and to explore the lack of any treatment effect for the combination of Aquamin and Glucosamine Sulfate in this small pilot trial. Although these products are unlikely to have reacted in the tablet form it is interesting to speculate about a possible dietary interaction, possibly related to the very basic nature of Aquamin (pH 10) compared to the acidity of the Glucosamine Sulphate (pH of 3.5 to 5), and the requirement for this to ionize in the stomach to be effective.

Aquamin is composed of multiple minerals and the 'active ingredient' for the complex is difficult to determine. A number of the minerals in Aquamin may have anti-inflammatory and anti-oxidant properties which might directly and/or indirectly influence the efficacy of this unique complex [13,14,16]. While the prominent mineral present in Aquamin is calcium (dosage = 80% Ca U.S RDA), its role in joint health is unclear. Magnesium however, was given at the daily dosage providing 14% (male) to 18% (female) U.S. RDA [12] and over the course of this study, this increased consumption of magnesium may have influenced OA symptoms by affecting the utilization of calcium or by potentially reducing inflammation around the affected joint. Both manganese and selenium were given at the daily dosage providing up to 16% and 4% of their RDA respectfully. Both of these trace minerals have been reported to reduce the appearance of osteoarthritic lesions and reduce the severity of symptoms in OA [14,16].

These pilot trial results suggest a potential treatment effect for Aquamin among subjects with moderate to severe OA and this preliminary finding warrants further study.

## Competing interests

Marigot Ltd. provided funding for this clinical trial and the article processing charges to publish this work. Melanie Walsh is a paid employee of Marigot Ltd., the sponsor of this work and provided only medical writing support for this manuscript. The other authors declare that they have no other competing interests. Marigot Ltd approved the protocol and reviewed the manuscript before submission for publication and can be reached at: Strand Farm, Currabinny, Carrigaline, Co. Cork, IRELAND; Phone: 353-21-437-8727; Fax: 353-21-437-8588. Marigot Ltd did not participate in any of the data collection or statistical analyses reported herein.

## Authors' contributions

JLF and MW co-authored the manuscript. JLF co-authored the protocol and directed the research team at MARC during the conduct of this trial. JLZ provided critical review of the manuscript, co-authored the protocol and provided medical monitoring services during the trial. MAK provided critical review of the manuscript and the protocol and provided statistical services for the design, execution and analysis of the data in this trial. All authors have read and approved the final manuscript.
